# Consultation with Quacks

**Published:** 1836

**Authors:** 


### ANALYTICAL.

### Art. I.—Consultation with Quacks—Puffing of Quack Medicines.

At a recent meeting of the Medical Council of the State Medical Society of Massachusetts, Dr. I. S. Bartlett was cited to appear to answer certain charges preferred against him for a violation of an article of the By-Laws of the Society inhibiting its members from any encouragement of empiricism. Dr. B. it seems had not only consulted with persons of bad reputation in medicine, but had puffed some of their nostrums. We notice this particularly with reference to the several modes in which regular practitioners in medicine may lend an effective aid to the dark deeds of medical imposture, by the line of professional conduct which they may pursue towards quacks and quack remedies. In the first place, educated and high minded physicians sometimes patronize quackery, by the eulogies bestowed by them upon quack medicines. All nostrums, or secret remedies, are to be regarded as such, in a correct estimate of charlatanry. He who advertises his remedies and cures, is essentially a quack, whatever pretensions he may otherwise make. Thus all quack remedies, specifics, panaceas, catholicans, etc., are in the just legitimate sense of the term quacks. This is the most indubitable evidence of empiricism. No man can escape the just application of the term quack, who deals in secret remedies. The physician who does so has the seal of condemnation put upon his character by the enlightened spirit of medical science. To aid such a course is next in degree of criminality to being a first perpetrator of the act. Guilt varies in degree in all moral aberrations. And the guilt of

*encouraging* quackery may vary likewise in degree. To be a quack—in an active and effective manner—a man must advertise his skill and cures—eulogise his remedies, and laud *his* many deeds of medical prowess. To praise the quack medicines of an ignorant presumptuous nostrum vender, is next in degree of criminal dereliction of professional morality, to being the original fabricator of the remedy, and of its ability to cure. For to fabricate cases of success with the new remedy is quite as common as to fabricate the nostrum itself.

To consult with a nostrum dealer is most assuredly a most effectual way of abetting quackery. When, therefore, we see the names of some of our most eminent Philadelphia and New York physicians, appended to the advertisements of Swaim—we have much to humble us in our views of the lofty position which American medicine should sustain in the eyes of the world.

"In folly's cup still laughs the bubble quackery;" Yes! and that bubble will forever laugh as long as there is ignorance to be duped, and credulity to operate upon. But should those who claim the title of honorable scientific physicians, in any obvious, demonstrable, willful manner, aid, abet, or instigate empirical acts? The answer which our readers will readily give is, no. Let then every one who sincerely honors his profession frown with indignant contempt upon every practitioner who deals in secret remedies—and never so far compromit his professional dignity as to mingle counsels with such men around the sick bed. Counsel! did we say—we retract the word. It is not in the nature of things possible for a nostrum vender to enter a sick chamber with any intention to deliberate and reflect upon the case to be submitted to his attention. Essentially vain in his pretensions of an infallible method of curing diseases the nostrum vender requires no light of symptomatology, or pathology to be reflected on the case. He can prescribe by intuition and accomplish his purposes in the mystery of a dark and deceptious legerdemain. We have never known these pretended superior remedies, circulated among the ignorant populace, to live long in the admiration of their own eulogists. One folly after another floats down the current, and each is caught at with avid. ity—till death engulphs both deceiver and deceived. In Louisville, Ky., several years ago an adventurer in physic, after entire failure in obtaining practice in a regular discharge of professional duties, gave out that he possessed a new remedy to destroy mercury in the system. He had a great run of practice, cheated many wiseacres by his pretended cures, and finally sunk into merited insignificance. His pretended anti-mercurial remedy, when made known by a student of his proved to be calomel (41 grs.)–rhubarb (8 grs.) and opium, (2 grs.) With this combination he salivated his patients severely, and this brought the mercury out of their bones. And yet this miserable ignorant impostor in medicine, was met in consultation by some, who considered themselves entitled to the appellation of scientific physicians. Nor do we deny that their general course as physicians fully entitled them to be so considered. But in consulting with a low minded nostrum vender they certainly sacrificed the high standard they once occupied as consistent and enlightened promoters of the dignity and usefulness of the medical profession. No compromise should ever be made with those pretenders to secret remedies, who encouraged by this ready way, afforded by ignorance, dishonesty, and impudence to accumulate money without intellectual or physical toil, are starting up, like exhalations from an unwholesome marsh, all around us.

Let every conscientious, enlightened practitioner rebuke, by his example as well as language, all such vile impositions, as the nostrum vender is daily perpetrating upon society. In thus discountenancing quackery he will subserve the interests of medical science, and his own self respect will rise in a correspondent degree to the noble disinterestedness and humane feelings, by which he is actuated in the cultivation and practice of his profession.

Since writing the above, we have received, the 19th No. (June 15th) of the Boston Medical and Surgical Journal, which contains an account of a meeting of the Boston Medical Association, held May 27th: Dr. Jacob Bigelow was in the chair. "The Secretary (Dr. Storer) read the record of doings at the annual meeting, concluding with the vote referring the charges against John S. Bartlett, M. D., to the Standing Committee. Also the proceedings of that committee at a meeting, held May 18th, at the house of Dr. George Hayward, at which Dr. Ware was appointed a sub-committee to communicate with Dr. Bartlett, and a resolution providing that if Dr. Bartlett did not make a proper apology, on or before the present meeting of the Association, he be expelled."

Dr. Ware reported that he had made a written communication to Dr. Bartlett, and also had a personal interview with him—that after a full discussion of the matter between them, Dr. Bartlett manifested no disposition to meet the wishes, or accede to the proposals, of the committee. Dr. Bartlett was present, and was heard in his own defence.—After a full and patient hearing of the Doctor, the Association, by a vote of 45 to 5, expelled Dr. John C. Bartlett from their number. The Massachusetts Medical Society had previously expelled him from their body. H.

